# Calpain-1 and Calpain-2 Promote Breast Cancer Metastasis

**DOI:** 10.3390/cells14171314

**Published:** 2025-08-25

**Authors:** Danielle Harper, Jung Yeon Min, James A. MacLeod, Samantha Cockburn, Iryna Predko, Yan Gao, Peter A. Greer, Ivan Shapovalov

**Affiliations:** 1 Department Pathology and Molecular Medicine, Queen’s University, Kingston, ON K7L3N6, Canada; danielle.harper@queensu.ca (D.H.); greerp@queensu.ca (P.A.G.); 2 Division of Cancer Biology and Genetics, Sinclair Cancer Research Institute, Queen’s University, Kingston, ON K7L3N6, Canada

**Keywords:** calpain, metastasis, breast cancer, CRISPR-Cas9, mouse model

## Abstract

In breast cancer, progression from localized stage I to distant metastatic stage IV disease is associated with a reduction of 5-year survival from nearly 100% to 23.2%. Expression of the calcium-activated protease isoforms calpain-1 and calpain-2 has been correlated with cell migration and invasion in vitro, metastatic potential in preclinical mouse models of cancer, and breast cancer prognosis in patients. It is unclear which of these two calpain isoforms is responsible for the apparent metastatic potential of cancer cells. Here, we demonstrate that while individual CRISPR-Cas9 knockouts of either *CAPN1* or *CAPN2* genes (encoding the catalytic subunits of calpain-1 and -2, respectively) reduce in vitro migration and marginally suppress in vivo metastasis, genetic disruption of both calpain-1 and calpain-2 through knockout of the *CAPNS1* gene (encoding the common regulatory subunit of calpain-1 and -2) diminishes metastasis by 83.4 ± 13.6% in a mouse xenograft model of human triple-negative breast cancer. The effect of calpain-1/2 deficiency was replicated in vitro with a modified cell-permeable calpastatin (CAST)-based peptide inhibitor (cell migration reduced to 53.5 ± 11.0% of vehicle control). However, this peptide inhibitor was not effective in vivo at reducing metastasis under the conditions used (vehicle vs. CAST, 1.12 ± 1.35 lung metastases per mm^2^ vs. 0.34 ± 0.20 metastases per mm^2^), likely due to rapid clearance, as indicated by the short serum half-life. This work demonstrates that calpain-1/2 disruption effectively abrogates metastasis and provides rationale for development of effective calpain inhibitors.

## 1. Introduction

Metastatic disease is the leading cause of death across a multitude of cancers, including breast cancer (BC) [1,2,3]. BC patients diagnosed with stage I disease have a near 100% 5-year overall survival rate, a 91.9% survival rate at stage II, and a 74.0% survival rate at stage III [4]. However, if distant metastasis is present (stage IV), survival rates drop to 23.2% [4]. Preventing the progression of early-stage cancer to metastatic disease or suppressing further metastatic spread in later-stage cancer may ameliorate this reduction in survival.

Metastatic dissemination may be prevented through disruption of cellular mechanisms required for cancer cell motility, invasion, or survival. Calpain proteases are promising therapeutic targets based on their mechanistic roles in each of these metastasis-promoting processes. The human calpain family consists of 15 isoforms, with calpain-1 and calpain-2 (calpain-1/2) being the first to be discovered [5,6]. Calpain-1/2 are ubiquitously expressed heterodimers consisting of a large catalytic subunit, encoded by *CAPN1* or *CAPN2*, respectively, and a small regulatory subunit encoded by *CAPNS1*. The regulatory subunit is required for catalytic subunit stability, and genetic disruption of *CAPNS1* is associated with a loss of both calpain-1 and calpain-2 activity [7].

Translational studies have reported that increased calpain-1/2 expression correlates with worse outcomes in BC [8], and pro-tumorigenic roles for calpains have emerged across various cancer types. For example, high *CAPNS1* expression is associated with metastasis and shorter survival in gastric cancer patients [9]. In pancreatic cancer, which is notorious for rapid metastatic spread, high tumor calpain-1 expression is associated with increased metastasis and shorter overall survival [10]. High calpain-2 expression in ovarian cancer has been linked to resistance to platinum-based chemotherapies, highlighting its potential use as a predictive biomarker [11]. Meta-analysis of data from several cancer types demonstrates a multi-fold increase in pro-metastatic features of tumors expressing high quantities of calpain-1/2 [12]

**Figure 1:**
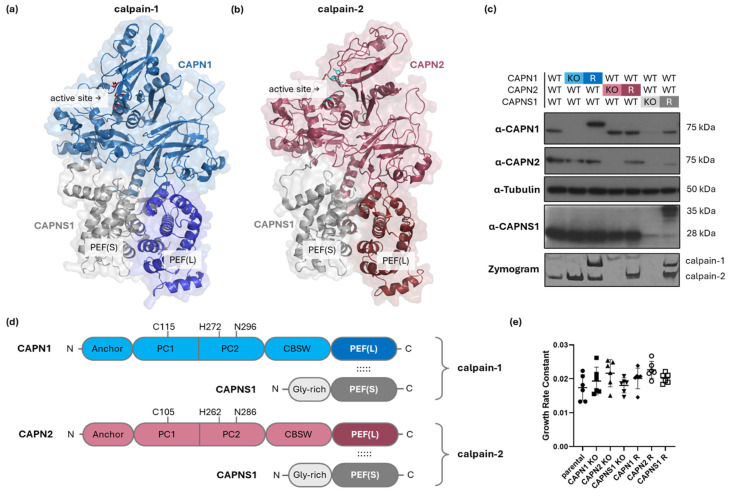
Genetic manipulation of *CAPN1, CAPN2*, and *CAPNS1* in MDA-MB-231 human TNBC cells. (**a**,**b**,**d**) Structures of calpain-1 and calpain-2. The model of (**a**) calpain-1 is based on an AlphaFold3 prediction (glycine-rich domain of CAPNS1 omitted) [16], while the model of (**b**) calpain-2 is based on a crystal structure PDB ID: 1KFU [15]. Both are heterodimers consisting of a common CAPNS1 regulatory subunit and CAPN1 or CAPN2 isoform-specific catalytic domains, respectively. The active site and heterodimerization-mediating PEF domains are indicated. (**c**) Immunoblotting analysis of parental MDA-MB-231 wild type (WT) and the respective knockout (KO) and rescue (R) cell lines with the indicated antibodies and tubulin, with casein zymography protease activity analysis shown in the bottom panel. (**d**) Domain maps of calpain-1/2 show the N-terminal anchor helix; PC1—protease core 1 domain; PC2—protease core 2 domain; CBSW—calpain-type beta-sandwich domain; and PEF(L)—penta EF-hand domain in the catalytic large subunits, CAPN1/2; and the Gly-rich—glycine-rich domain; and PEF(S)—penta EF-hand domain in the regulatory small subunit, CAPNS1. Amino acids of the catalytic triad are indicated. The dotted lines indicate interactions of PEF(L) and PEF(S) mediating dimerization of CAPN1/2 with CAPNS1. (**e**) Cell growth rates of the indicated cell lines. Across all genotypes, no significant differences in proliferation rate were observed (one-way ANOVA, F(6,35) = 2.079, *p* = 0.0809).

Calpain-1/2 isoforms contribute to tumor cell migration, invasion, and metastasis by limited proteolysis of intracellular protein substrates, including those which contribute to the regulation of focal adhesion dynamics and promotion of cytoskeletal remodeling (reviewed in [13]). However, our understanding of which calpain isoforms are responsible for these metastasis-promoting functions, and through which substrates, is incomplete since there is evidence for calpain-1 and calpain-2 cleaving either redundant or isoform-specific substrates. Structurally, calpain-1 and calpain-2 are very similar (Figure 1a,b,d), sharing 53% amino acid identity and 78% amino acid similarity (sequence alignment shown in Supplementary Information 1), suggesting comparable structure–function attributes [14,15].

Calpain-1-specific substrates include epidermal growth factor receptor (EGFR) [17] and human epithelial growth factor receptor 2 (HER2) [18]. Calpain-1 also plays a unique role in the cleavage and inactivation of the RhoA GTPase, a key player in the formation of stress fibers and focal adhesion complexes [19]. Calpain-2 has been specifically implicated in the proteolysis of the cytoskeletal protein talin, which is required for focal adhesion disassembly [20], as well as the cleavage of focal adhesion kinase (FAK) [21], which regulates focal adhesion dynamics, in part by altering FAK interactions with components of focal adhesion [22]. Calpain-2-specific cleavage of filamin A (FLNA) has been shown to produce a C-terminal fragment that mediates nuclear translocation of the HIF1α transcription factor [23]. These observations suggest isoform-specific functions for calpain-1 and calpain-2. At the same time, there are several calpain substrates, for example, E-cadherin [24], c-Myc [25], Bax [26], and spectrin [27,28], which can be cleaved by either calpain isoform, or for which isoform specificity is not apparent.

Due to such complexity in the functions of calpain-1 and calpain-2, it is not clear whether these isoforms play unique or redundant roles in metastasis. To study this, we employed CRISPR-Cas9 gene editing [29] to manipulate calpain-1/2 expression in the highly metastatic human MDA-MB-231 triple-negative breast cancer cell line.

Commercially available calpain inhibitors lack calpain selectivity, not to mention calpain isoform specificity. To partially circumvent these issues, we employed a cell-permeable inhibitory peptide, derived from the endogenous calpain-1/2 inhibitor calpastatin (CAST), as a direct pseudo-pharmacological approach to inhibit calpain-1/2 activities. This CAST peptide specifically binds and blocks the catalytic cleft of calpain-1/2, enabling us to mimic our genetic knockouts in vitro and confirm that suppression of calpain-1/2 activities in vivo attenuates the metastatic phenotype of cancer cells.

To study calpain-dependent metastasis, using the lentiCRISPRv2 CRISPR-Cas9 knockout (KO) system [29], we tested the effect of individual *CAPN1* or *CAPN2* KOs in the human triple-negative breast cancer cell line MDA-MB-231, or disruption of both calpain-1/2 through KO of *CAPNS1*, encoding the common regulatory subunit which renders both catalytic subunits inactive and unstable [7]. We also rescued expression of *CAPN1*, *CAPN2*, and *CAPNS1* in the respective KO cell lines using lentiviral vectors to confirm that the KO phenotypes were associated with loss of calpain-1/2. Immunoblotting and casein zymography analysis [30] confirmed KO and rescue (R) of calpain subunit expression and proteolytic activity, respectively. This panel of *CAPN1*, *CAPN2*, and *CAPNS1* KO and R cell lines was used to explore the role of calpain-1/2 isoforms in cancer cell migration and invasion in vitro, including velocity and directionality [31], and in vivo tumor growth and metastasis using an orthotopic mouse engraftment model (similarly to [32]) in *Rag2^-/-^ IL2Rγc^-/-^* mice [33].

## 2. Materials and Methods

### 2.1. Tissue Culture: CRISPR-Cas9 Gene Knockout and Rescue

All tissue culture procedures were performed in a laminar flow hood in a level 2+ certified laboratory. HEK293T (ATCC # CRL-3216) and MDA-MB-231 (ATCC # HTB-26) cells were cultured in TC-treated 10 cm plates (Sarstedt, Nümbrecht, Germany) in phenol red containing Dulbecco’s Modified Eagle Medium (Life Technologies, Carlsbad, CA, USA), supplemented with antibiotic–antimycotic mix (100 units/mL penicillin and 100 µg/mL streptomycin, Life Technologies, Carlsbad, CA, USA), 2 mM L-glutamine (Mediatech, Manassas, VA, USA), 10 μg/mL ciprofloxacin (GenHunter, Nashville, TN, USA), and 10% Fetal Bovine Essence (Avantor Seradigm, Radnor Township, PA, USA) (complete DMEM) at 37 °C and 5% CO_2_ in a ThermoForma incubator (ThermoFisher Scientific, Waltham, MA, USA).

CRISPR-Cas9 gene KOs were achieved by transducing MDA-MB-231 cells with lentivirus produced in HEK 293T cells transfected with 6 μg psPAX2 and 2 μg pMD.2G packaging plasmids, along with 8 μg sgRNA-expressing LentiCRISPRv2 [29] plasmids in TC-treated 10 cm plates. The DNA-encoding gene-targeting sgRNA sequences which were cloned into the BsmBI site of LentiCRISPRv2 and their intended target sequences are shown in Supplementary Information 2–6. The deoxyoligonucleotides used to generate these gene-targeting vectors are shown in Supplementary Information 3. Lentivirus-transduced MDA-MB-231 cells were selected with 2 μg/mL puromycin, and the success of gene KO was assessed by immunoblotting polyclonal populations for the proteins of interest with the respective antibodies to CAPN1, CAPN2, or CAPNS1, followed by cloning and immunoblotting validation of selected clones. The CRISPR-Cas9-induced mutations in individual clones were confirmed by PCR followed by Sanger sequencing (Robarts Research Institute, London, ON, Canada).

Gene rescues in KO clones were achieved with lentiviruses containing cDNAs engineered with silent mutations designed to protect the integrated rescue lentiviral genomes from CRISPR-Cas9 targeting. The human *CAPN1, CAPN2*, and *CAPNS1* cDNAs were first PCR-amplified from reverse-transcribed RNA isolated from MDA-MB-231 cells, cloned into the NcoI site of the pCS2-MT expression vector to incorporate an in-frame Myc-epitope tag on their N-termini (for *CAPN1* and *CAPNS1* only), and then PCR-cloned into the PmeI site of the pWPXLd lentiviral expression vector with an internal ribosome entry site (IRES) and GFP open reading frame after the calpain open reading frame. Deoxyoligonucleotides used to generate these rescue constructs are shown in Supplementary Information 4 and Supplementary Information 5. Silent mutations were engineered into these *CAPN1*, *CAPN2*, and *CAPNS1* rescue constructs to destroy the PAM sequence or sgRNA-homologous sequences used in the knockout, as shown in Supplementary Information 6. These mutations were generated using the QuikChange II XL Site-Directed Mutagenesis Kit according to the manufacturer’s instructions (Agilent, Santa Clara, CA, USA). Rescue KO cells were established by transfection of KO clones with lentivirus produced in HEK 293T cells transfected with 6 μg psPAX2, 2 μg pMD.2G, and 8 μg of the respective pWPXLd plasmids in TC-treated 10 cm plates. For engraftment studies, a similar secondary transduction with a GFP-expressing pWPXLd lentivirus was used to obtain green fluorescent MDA-MB-231 cells.

Plasmid transfections to make lentivirus were conducted in HEK 293T cells using PolyJet transfection according to the manufacturer’s protocol (SignaGen Frederick, MD, USA, Cat# SL100688). Virus was collected 24 and 48 h after transfection, 0.45 µm filter-sterilized, and used to transduce cells in the presence of polybrene. KOs were confirmed by immunoblotting and sequencing of individual clones. Polyclonal rescues of each KO were confirmed by immunoblotting.

### 2.2. SDS-PAGE Immunoblotting

Polyacrylamide gels were made with 29:1 acrylamide/bis-acrylamide (ThermoFisher Scientific, Waltham, MA, USA), Tris-HCl (ThermoFisher Scientific, Waltham, MA, USA)-based buffers, 10% ammonium persulfate (ThermoFisher Scientific, Waltham, MA, USA), and TEMED (Sigma-Aldrich, St. Louis, MO, USA). Cell lysates were made in kinase lysis buffer (20 mM Tris-HCl pH 7.5, 150 mM NaCl, 1 mM EDTA, 1% *v*/*v* Nonidet P-40, 0.5% *w*/*v* sodium deoxycholic acid), supplemented with 100 µM PMSF, 10 ng/mL leupeptin, 100 µM aprotinin, and 100 µM sodium orthovanadate, prepared as 5:1 lysate/6X Laemmli sample buffer mixtures. The gels were run at a constant 30 mA per gel and ≤150 V. BLUelf prestained protein markers (FroggaBio, Concord, ON, Canada) were used to estimate molecular weights. The resulting gels were transferred onto a PVDF membrane and immunoblotted with antibodies to CAPN1, CAPN2 (#2556 and #2539, respectively, Cell Signaling Technology, Boston, MA, USA), or CAPNS1 (sc-32325, Santa Cruz Biotechnology, Dallas, TX, USA) at 1/1000 dilution in 5% BSA in TBS-T at 4 °C overnight, followed by secondary anti-rabbit or anti-mouse HRP conjugates (#7074 or #7076, respectively, Cell Signaling Technology, Boston, MA, USA) at 1:2500 dilution in 5% *w*/*v* BSA in TBS-T at room temperature for 1 h. Detection was carried out using Western Lightning Plus ECL kit HRP reagents according to the manufacturer’s instructions (PerkinElmer, Shelton, CT, USA) with 100 NIF Fuji Medical X-ray film (Fujifilm, Tokyo, Japan) and an SRX-101A medical film processor (Konica Minolta, Tokyo, Japan).

### 2.3. Casein Zymogram

Composition of reagents for casein zymography is shown in Supplementary Information 7. Gels, lysis buffer, and running buffer for HEPES–Imidazole casein zymography were prepared as described [30]. After electrophoresis, gels were incubated in 100 mL development buffer for 16 h, fixed in 100 mL 1:1:8 acetic acid/methanol/water for 15 min, stained with 100 mL fix buffer containing 0.1% (*w*/*v*) Coomassie Blue for 30 min, and destained in 250 mL 1:1:8 acetic acid/methanol/water overnight. The resulting gel was imaged with the FluorChem imager (Alpha Innotech, San Leandro, CA, USA).

### 2.4. Incucyte Cell Growth Rate Assay, Video Microscopy, and Spider Graph Migration Analysis

Cells were seeded in 96-well ImageLock plates (Essen Bioscience, Ann Arbor, MI, USA) at 4000 cells per well and incubated at 37 °C and 5% CO_2_ overnight. The next day, the plates were imaged using the Incucyte Live-Cell Analysis System (Essen Bioscience, Ann Arbor, MI, USA) every 2 h for 24 h. A generic Incucyte confluency measurement algorithm was used to analyze the images [34]. The resulting growth curves were log2-transformed, and the growth rate constants were defined and measured as linear slopes of the resulting curves. Growth rate constants are presented as mean ± standard deviation of multiple replicate measurements.

For the migration assay, plates were coated with 15 µg/mL rat tail collagen type 1 (BD-Biosciences, Franklin Lakes, NJ, USA). Cells were seeded at 1000 cells per well. Plates were imaged using the IncuCyte Live-Cell Analysis System (Essen Bioscience, Ann Arbor, MI, USA) every 10 min for 24 h. Cell migration was tracked using the “Track Object” program in Metamorph 7.8 (Molecular Devices, LLC., San Jose, CA, USA). The resulting coordinates were analyzed with the DiPer program [31] to measure speed and directional persistence of migration [31]. Experiments were conducted in triplicate, each with at least 20 cells per genotype. The results are presented as the mean ± standard error of the mean.

### 2.5. Orthotopic Engraftment Model of Mammary Tumorigenesis

Cells were washed, counted, and resuspended in PBS to achieve a concentration of 1,000,000 cells in 40 µL and placed on ice and supplemented with Growth Factor Reduced Matrigel (Corning Inc., Corning, NY, USA, catalog number 354,230) in a 10:40 Matrigel/cell suspension ratio. The resulting mixture was maintained on ice until the engraftment. Matrigel was included in the engraftment suspension to promote initial cell retention in the engrafted bolus in the mammary fat pad and to ensure consistent tumor onset. While MDA-MB-231 cells can form tumors without matrix in *Rag2^-/-^ IL2Rγc^-/-^* mice, the addition of Matrigel could reduce experimental variability and mimic the basement membrane or extracellular matrix components of the tumor microenvironment.

All animal procedures were approved by the university animal care committee in accordance with guidelines from the Canadian Council on Animal Care. Experiments were conducted on 12–14-week-old female *Rag2^-/-^ IL2Rγc^-/-^* mice [33]. Mice were anesthetized with isoflurane, and incisions were made to expose the #4 inguinal mammary fat pad. Using a 50–100 µL Hamilton syringe, 50 µL of Matrigel/cancer cell suspension was injected into the mammary fat pad. The incision was closed with 9 mm wound clips (BD cat:22–275998), and the animals were administered 2 mg/kg meloxicam as an analgesic and 0.5 mL sterile PBS to compensate for fluid loss and allowed to recover. The analgesic was administered daily for two additional days after surgery.

The wound clips were removed one week after the engraftment surgery, and the tumor size was measured daily until it reached 600 mm^3^. For tumor growth curves, the time of tumor onset was established as the point when tumors reached 2 mm in diameter, and the tumor growth rate was defined as a linear slope of the growth curve between 2 mm and 10 mm in diameter. These values are presented as mean ± standard deviation. When tumors reached the maximum volume (19 to 22 days after the engraftment surgery, determined individually for every mouse), they were resected by recovery surgery, and mice were then monitored for 12 days before being euthanized with CO_2_ and cervical dislocation and assessed for metastasis. The pericardial cavity was opened to expose and remove the lungs, which were washed in PBS and placed in 35 mm dishes for fluorescent imaging.

### 2.6. Biophotonic Imaging

Organs were imaged with the GFP Ex/Em setting on an EVOS7000 microscope (ThermoFisher Scientific, Waltham, MA, USA). ImageJ 2.17.0 software (National Institutes of Health, Bethesda, MD, USA) was used to measure total % tumor burden and to count individual metastatic lesions. The values from posterior and anterior images were averaged to obtain a single value for each animal. The values for each cohort are presented as the mean ± standard deviation. Experiments for *CAPN1* KO/R, *CAPN2* KO/R, and *CAPNS1* KO were conducted in duplicate, with at least 3 mice per genotype. A total of 9 mice were engrafted with *CAPNS1* R cells within one experiment. Each experiment included ≥5 mice within the WT control group.

### 2.7. Peptide Synthesis and Assays

Wild-type and modified B27 peptides were synthesized at 75% purity by GenScript (Nanjing, China). The in vitro cell migration assays were conducted at 4 µM peptide concentration. This experiment was performed once with 60 cells per experimental group. The in vivo mouse study was conducted as described above, with mice treated intraperitoneally with the mB27 peptide at 5 mg/kg/day for the duration of tumor growth from the initial volume of 50 mm^3^ to a final volume of 600 mm^3^. Data represents one experiment with 9–10 mice per group.

### 2.8. Data Analysis

Statistical analyses, linear and non-linear curve fitting, and data visualization were performed with GraphPad Prism 10 (Boston, MA, USA). Protein models were obtained via PDB or generated with AlphaFold 3 (Google DeepMind, London, UK) [16] and visualized with the PyMOL Molecular Graphics System, version 2.5.2, Schrödinger, LLC (New York, NY, USA). Statistical significance was defined as *p* < 0.05 (*), *p* < 0.01 (**), *p* < 0.001 (***), and *p* < 0.0001 (****).

For the sample size calculations for the in vivo metastasis experiments, the defined false-positive rate (alpha error) was 0.05 and the false-negative rate (beta error) was 0.2. The standardized wild-type metastasis produced an error of ±56%. Therefore, to observe an anticipated difference of one standard deviation (100% WT vs. 32% KO), the required sample size (n) was 16 mice in the WT cohort and 8 mice for each KO cohort. The actual sample sizes were 29, 8, 11, and 9 mice for the WT, *CAPN1* KO, *CAPN2* KO, and *CAPNS1* KO cohorts, respectively.

## 3. Results

### 3.1. CRISPR-Cas9 Knockout of CAPN1, CAPN2, or CAPNS1 Effectively Abolishes Calpain Expression and Activity

Currently, no commercial inhibitors can specifically target calpain-1 or calpain-2, making it difficult to study calpain deficiency or distinguish between the two isoforms. Therefore, we have generated a panel of CRISPR-Cas9 KO MDA-MB-231 triple-negative breast cancer cell lines for *CAPN1*, *CAPN2*, or *CAPNS1* genes, which provide models for complete pharmacological inhibition of calpain-1, calpain-2, or both, respectively. Rescues of each KO line were also generated by lentiviral transduction with cDNA transgenes to verify the specificity of phenotypes produced in the knockouts. Knockout and rescue strategies are described in the Materials and Methods Section, with additional details provided in Supplementary Information 2–6.

Immunoblotting for CAPN1, CAPN2, and CAPNS1 confirmed ablation of protein expression in the cloned KO cell lines and reintroduction of expression in the polyclonal rescue (R) lines (Figure 1c). Casein zymography [30] demonstrated proteolytically active forms of calpain-1 and calpain-2 (or their absence) in the corresponding cell lines (Figure 1c, bottom panel). Note that CAPN1 and CAPNS1 in cell lines expressing the rescue constructs migrate slower than their endogenous counterparts due to the incorporation of Myc-epitope tags. The corresponding calpain-1 and calpain-2 activities detected in the zymography analysis of these rescue cell lines also display slightly different migration.

Because calpains are known to affect proliferative and apoptotic pathways in certain contexts [35], we tested the effect of calpain-1/2 deficiency on the proliferation rates of the cell lines using timelapse microscopy. None of the genetic alterations significantly affected proliferation rates in the panel of cell lines (Figure 1e).

### 3.2. Loss of Either Calpain-1 or Calpain-2 Impairs Cell Migration, and Loss of Both Produces the Strongest Defect

Migration propensity of calpain-deficient cells was measured by IncuCyte video microscopy of single-cell trajectories on a collagen substrate, which were then analyzed according to an established protocol using three measures of migration [31]: (1) speed of migration (a raw value of displacement from frame to frame); (2) directionality (a ratio of cell displacement to path); and (3) mean square displacement (MSD, how much area a migrating cell has covered).

*CAPN1* KO cells exhibited reduced cell migration speed (90.25 ± 1.8% of WT control), a reduced directionality ratio at the last point of the trajectory (0.20 ± 0.01 KO vs. 0.24 ± 0.01 WT), and a reduced mean square displacement (75.3 ± 14.1% of WT control at the last point of the trajectory). All three phenotypes were recovered with the CAPN1 rescue (99.2 ± 2.2%, 0.28 ± 0.01, and 120.8 ± 18.1%, respectively). The *CAPN1* KO/R effects on speed, directionality, and MSD of migration were statistically significant (one-way ANOVA F[2,148] = 7.428, *p* = 0.0008; one-way ANOVA F[2,148] = 10.83, *p* < 0.0001; and ANCOVA F[1,2196] = 11.05, *p* = 0.0009, respectively).

Similarly, *CAPN2* KO cells exhibited reduced cell migration speed (75.9 ± 1.8% of WT control), a reduced directionality ratio at the last point of the trajectory (0.17 ± 0.01 vs. 0.24 ± 0.01 WT), and reduced mean square displacement (39.2 ± 14.9% of WT control at the last point of the trajectory). All three phenotypes were recovered with the CAPN2 rescue (117 ± 14.6%, 0.24 ± 0.01, and 80.4 ± 14.6%, respectively). The CAPN2 KO/R effects on the speed, directionality, and MSD of migration were statistically significant (one-way ANOVA F[2,169] = 6.541, *p* = 0.0018; one-way ANOVA F[2,169] = 11.10, *p* < 0.0001; and ANCOVA F[1,2548] = 50.81, *p* < 0.0001, respectively).

And finally, *CAPNS1* KO cells, which lack both calpain-1 and calpain-2, exhibited reduced cell migration speed (78.1 ± 1.5% of WT control), a reduced directionality ratio at the last point of the trajectory (0.15 ± 0.01 vs. 0.24 ± 0.01 WT), and reduced mean square displacement (20.1 ± 2.1% of WT control at the last point of the trajectory). All three phenotypes were recovered with the *CAPNS1* rescue (108 ± 2.6%, 0.22 ± 0.01, and 123.7 ± 22.0%, respectively). The *CAPNS1* KO/R effects on the speed, directionality, and MSD of migration were statistically significant (one-way ANOVA F[2,158] = 64.63, *p* < 0.0001; one-way ANOVA F[2,158] = 17.04, *p* < 0.0001; and ANCOVA F[1,2438] = 223.9, *p* < 0.0001, respectively). A thorough quantification and statistical analysis of cell migration is presented in Supplementary Information 8.

**Figure 2:**
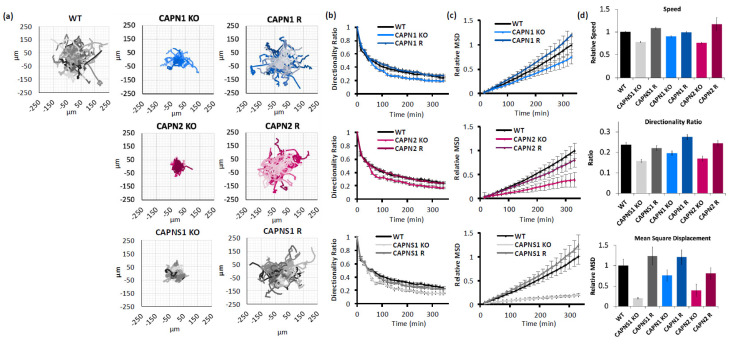
Calpain-1 and calpain-2 contribute to MDA-MB-231 cell motility. (**a**) Spider graphs, (**b**) directionality ratios, and (**c**) relative mean square displacement of trajectories of migrating MDA-MB-231 cells of the indicated genotypes; (**d**) average speed, directionality, and MSD of cells at the last point of the trajectory. Data represents 140 cells per genotype across 3 experiments.

In summary, isoform-specific KO of either *CAPN1* or *CAPN2* produced a migration defect, seen in multiple measures, which was successfully rescued by the gene addback (Figure 2). The double knockout of calpain-1 and calpain-2, through *CAPNS1* KO, produced the greatest migration defect, suggesting non-redundant roles for calpain-1 and calpain-2 in cell-intrinsic functions required for cell migration, including focal adhesion turnover, actin remodeling, and leading/trailing-edge membrane–cytoskeletal dynamics.

### 3.3. Loss of Both Calpain-1 and Calpain-2 Significantly Reduces Breast Cancer Metastasis in a Mouse Model

**Figure 3:**
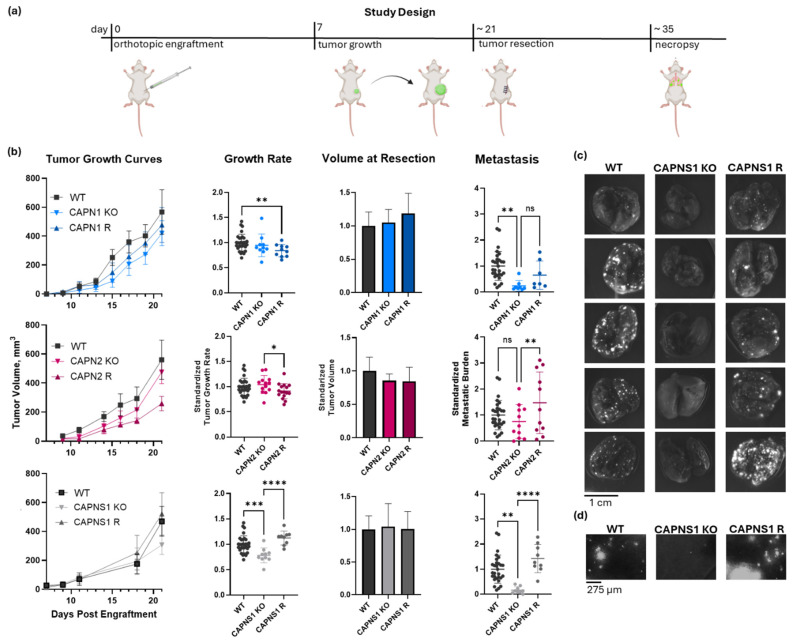
In vivo metastasis of *CAPN1*, *CAPN2*, and *CAPNS1* KO and R MDA-MB-231 human TNBC cells in an orthotopic engraftment model in *Rag2^-/-^IL2Rγc^-/-^* mice. (**a**) The resect-and-wait study design; (**b**) growth curves, growth rates, and standardized metastasis of WT (n = 29), *CAPN1* KO (n = 8), *CAPN1* R (n = 7), *CAPN2* KO (n = 11), *CAPN2* R (n = 11), *CAPNS1* KO (n = 9), and *CAPNS1* R (n = 9) tumors; (**c**) representative images of lungs of tumor-bearing mice with tumors of the indicated genotypes fluorescently imaged in a GFP channel (Ex/Em: 488/510 nm)—each hyperintense nodule is a GFP-positive metastatic lesion; (**d**) representative images of a GFP-channel 10× field of view of lungs of tumor-bearing mice with tumors of the indicated genotypes. The complete image dataset is available in the Zenodo data repository at https://doi.org/10.5281/zenodo.16790973 (version 1, 10 August 2025) [36]. Statistical significance was defined as *p* < 0.05 (*), *p* < 0.01 (**), *p* < 0.001 (***), *p* < 0.0001 (****), and ns: not significant.

Next, we conducted metastasis studies using a murine orthotropic engraftment model. These in vivo experiments assessed the panel of calpain-1/2 KO and R cells to establish whether the observed in vitro phenotypes correlated with a similar non-redundant isoform requirement for metastasis in vivo. Upon engraftment of 500,000 WT MDA-MB-231 cancer cells into the #4 mammary fat pad of *Rag2^-/-^IL2Rγc^-/-^*mice, as illustrated and described in Figure 3a, the resulting tumors grew at an average rate of 0.58 ± 0.15 mm diameter per day. When these tumors reached 600 mm^3^ in volume, they were resected, and the metastasis was assessed 12 days later, at which point an average WT control tumor-bearing mouse had 6.7 ± 3.7 micrometastases per 1.16 mm^2^ of lung area, which is a 10× field of view on the EVOS M7000 fluorescent microscope.

The tumor growth produced by the calpain-deficient tumor cells, shown in Figure 3b, was significantly different (one-way ANOVA F[6,92] = 5.419, *p* < 0.0001). *CAPN1* KO and *CAPN1* R tumors grew at 94.6 ± 22.5% and 84.0 ± 12.4% of the WT rate, respectively, where the KO was not significantly less than WT, but the rescue was significantly less than the WT (individual *p*-values of 0.366 and 0.009, respectively). *CAPN2* KO and R tumors grew similarly to the WT at 104.1 ± 18.0% and 91.0 ± 14.9% of the WT rate, respectively, and the differences were not significantly different from the WT (individual *p*-values of 0.457 and 0.080, respectively). The *CAPNS1* tumors did grow 22 ± 14.6% slower than WT (*p* = 0.001), but this was unlikely to affect metastasis outcomes since the tumors were resected at size endpoints rather than time endpoints. The *CAPNS1* R tumors were not significantly different from WT tumors (112 ± 13.7%, *p* = 0.051). A thorough quantification and statistical analysis of tumor growth rates are presented in Supplementary Information 9.

The metastasis produced by tumors is shown in Figure 3b and illustrated in Figure 3c,d. Metastasis was significantly different (one-way ANOVA F[6,77] = 6.294, *p* < 0.0001). *CAPN1* KO and *CAPN1* R tumors produced 23.2 ± 20.5% and 64.7 ± 54.5% as many metastatic events as WT tumors, respectively, where the KO was significantly less than WT, but the rescue was not significantly different from the KO (individual *p*-values of 0.004 and 0.215, respectively). *CAPN2* KO and *CAPN2* R produced 75.1 ± 65.0% and 147.6 ± 118% metastatic events relative to WT tumors, respectively, where the KO was not statistically distinguishable from the WT, but R was significantly greater than the KO (individual *p*-values of 0.277 and 0.010, respectively). Most notably, *CAPNS1* KO and *CAPNS1* R tumors produced 16.6 ± 13.6% and 142.6 ± 55.9% metastatic events relative to WT tumors, respectively, where the KO was significantly less than WT, and the R significantly restored metastasis relative to the KO (individual *p*-values of 0.001 and <0.0001, respectively). A thorough quantification and statistical analysis of metastasis for all cohorts are presented in Supplementary Information 10. The complete lung image dataset is available in the Zenodo data repository at https://doi.org/10.5281/zenodo.16790973 (version 1, 10 August 2025) [36].

In summary, the effects of individual isoform knockouts on metastasis were variable. *CAPN1* KO tumors exhibited significantly reduced metastasis compared to WT, whereas *CAPN2* KO tumors did not. In the rescue lines, *CAPN1* R metastasis was not statistically significantly restored relative to the KO, while *CAPN2* R metastasis was significantly increased compared to the KO. These results suggest that the loss of either isoform alone can produce partial or inconsistent suppression of metastasis, with possible differences in compensatory mechanisms or complicated by the stochastic nature of metastasis. In contrast, *CAPNS1* KO, which eliminates both calpain-1 and -2, consistently reduced metastasis by >80% and was fully rescued by *CAPNS1* re-expression (Figure 3b). Thus, we conclude that combined inhibition of both calpain-1 and calpain-2 is necessary to achieve a robust anti-metastatic effect in this orthotopic engraftment resect-and-wait model.

### 3.4. Calpastatin-Based Peptide Shows Limited Efficacy Against Calpain-Mediated Migration and Metastasis

**Figure 4:**
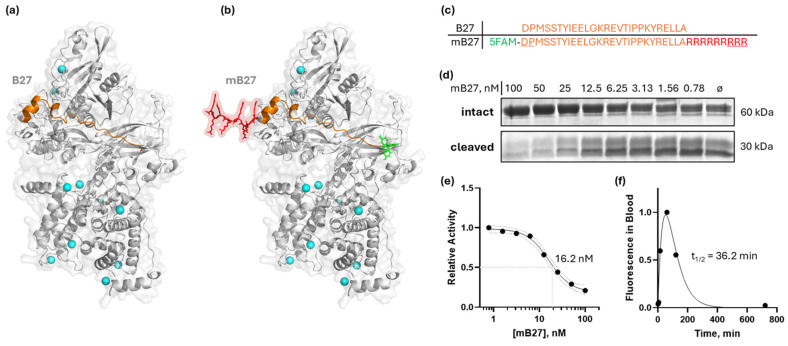
Design of a modified B27 peptide of CAST, mB27, for calpain inhibition. (**a**) An AlphaFold [16] model of B27 binding to the active site cleft of calpain-2; (**b**) an AlphaFold [16] model of a modified B27 peptide (mB27) binding to calpain-2; (**c**) primary sequences of B27 and mB27 (5FAM [5-carboxyfluorescein] in green; additional arginine residues to enable cell penetration in red; D-enantiomeric amino acids are underlined); (**d**) dose-dependent inhibition of calpain-2 by mB27, measured on a recombinant substrate as in [40]; (**e**) sigmoidal curve approximation of dose-dependent inhibition of calpain-2—IC_50_ = 16.2 nM (95% CI: 13.1 to 21.5 nM); (**f**) fluorescence of mB27 in mouse plasma measured at indicated times after intraperitoneal injection of 10 mg/kg mB27. The plasma half-life of mB27 fitted with a Bateman function was 36.2 min.

Aberrant expression and activity of calpain-1/2 isoforms have been implicated in cancer and other diseases. Consistent with these findings, we have provided genetic evidence that inhibition of both calpain-1 and calpain-2 has the potential to abrogate metastatic breast cancer. However, there are currently no clinically approved small-molecule calpain-1/2 inhibitors; the inhibitors that have been published suffer from low specificity for calpains, or selectivity against other cysteine proteases. These limitations complicate their use in preclinical in vivo studies. Therefore, to replicate the effects of the genetic KO, which mimics a 100% pharmacological inhibition, we instead employed a CAST peptide approach. Calpastatin, CAST, is a known endogenous calpain-1/2 isoform-specific inhibitor which binds with its A and C motifs to the PEF domains, and at the catalytic cleft with its B motif [37]. The 27-amino-acid sequence within the B motif (B27) that is responsible for most of the binding/inhibitory activity—DPMSSTYIEELGKREVTIPPKYRELLA [38]—is shown in Figure 4a,c, and it corresponds to amino acids 267–293 of human calpastatin isoform a (NCBI accession number NP_001741). The B27 peptide effectively inhibited recombinant calpain-2 (Supplementary Information 11). However, since natural peptides have poor membrane permeability, we chose to modify the B27 peptide with a membrane-penetrating oligo-arginine, similarly to [39]. Peptides suffer from poor pharmacokinetics due to degradation in the blood and liver, as well as kidney filtration. To ameliorate this, we modified the B27 peptide with D-amino acids at N- and C-termini to abrogate peptide cleavage in blood, and we also added an N-terminal 5/6-FAM fluorescent tag for peptide detection (Figure 4b,c). The resulting synthetic modified B27 (mB27) peptide maintained its in vitro calpain inhibition property, at 16.2 nM IC_50_ (95%CI 13.1 to 21.5 nM) (Figure 4d,e), on par with the reported values for the B27 peptide without N- and C-terminal modifications [38].

**Figure 5:**
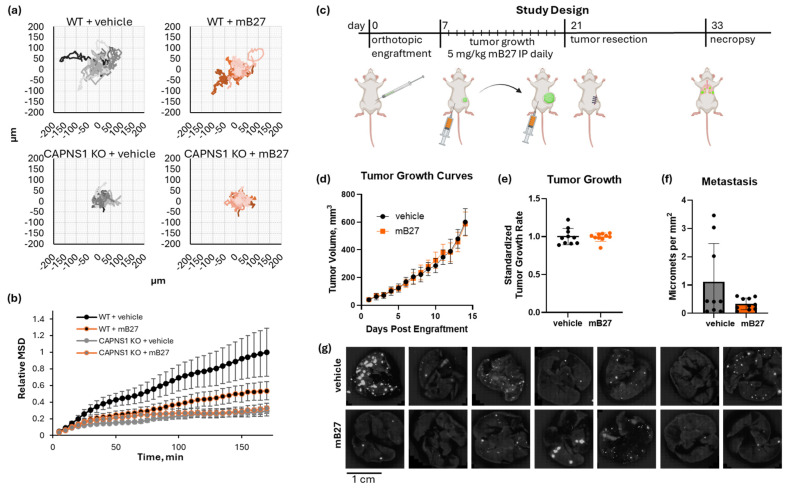
Inhibition of calpain by a modified B27 peptide of CAST. (**a**) Spider graphs of WT or CAPNS1 KO MDA-MB-231 cells migrating in the presence of 4µM mB27 or a vehicle control; (**b**) mean square displacement (MSD) of the trajectories in (**a**); (**c**) a resect-and-wait study design, where 500,000 WT MDA-MB-231 cells were orthotopically engrafted, and mice were treated with either 5 mg/kg/day mB27 (n = 10) or a vehicle control (n = 9) for 14 days starting at 50 mm^3^ tumor volume and ending at 600 mm^3^, followed by a recovery resection surgery, a wait for 12 days, and a necropsy analysis of lung metastasis. (**d**) Tumor growth curves, (**e**) tumor growth rates, and (**f**) quantified metastases for the resulting tumors; (**g**) representative images of lungs of tumor-bearing mice treated with either 5 mg/kg/day mB27 or vehicle control. The complete image dataset is available in the Zenodo data repository at https://doi.org/10.5281/zenodo.16790973 (version 1, 10 August 2025) [36].

To evaluate the effect of this calpain inhibitor, we first measured MDA-MB-231 cancer cell migration in the presence of 4 µM mB27 (Figure 5a,b). The peptide treatment reduced cancer cell migration to 53.5 ± 11.0% of the vehicle control, which was statistically significant (ANCOVA F[1,1356] = 18.32, *p* < 0.0001). In contrast, the peptide had no effect on the CAPNS1 KO cells, where the MSD values in the presence of the peptide or vehicle were 29.3 ± 5.8% and 32.5 ± 6.3% of the WT vehicle control, respectively (ANCOVA F [1,1356] = 0.2554, *p* = 0.6134). A thorough quantification and statistical analysis of cell migration are presented in Supplementary Information 12.

Since mB27 successfully inhibited cell migration in vitro, we next tested its ability to inhibit in vivo metastasis using the orthotopic engraftment model (Figure 5c). Tumor-bearing mice were treated once daily with 5 mg/kg mB27 or a vehicle control for 14 days starting at a 50 mm^3^ tumor volume and ending at 600 mm^3^. WT MDA-MB-231 tumors grew at an identical rate in both cohorts (mB27 vs. vehicle, 0.428 ± 0.024 mm diameter per day vs. 0.431 ± 0.046 mm diameter per day, respectively; unpaired *t*-test t [18] = 0.1849, *p* = 0.8554) (Figure 5d,e). Upon completing the resect-and-wait model, metastasis was evaluated (Figure 5f,g). Lung metastasis in the vehicle vs. mB27 cohorts was 1.12 ± 1.35 and 0.34 ± 0.20 metastases per mm^2^, respectively. The difference in the average metastasis was not statistically significant (Welch’s *t*-test, t[8.3] = 1.841, *p* = 0.102), but there was a large difference in variance (F[8,9] = 46.5, *p* < 0.0001), which indicates that there were significantly more high-metastasis outliers in the control group that were not observed in the mB27 treatment group.

## 4. Discussion and Conclusions

For almost 30 years, calpains have been considered as possible therapeutic targets in cancers [41]. The data presented in this study support the conclusion that calpain inhibition may offer therapeutic benefits for patients presenting with pre-metastatic breast cancer (stages I, II, III). In these circumstances, calpain inhibition might suppress progression to distant metastasis. Even when diagnosed early, increased wait times for surgery, chemotherapy, and radiotherapy provide an opportunity for tumors to progress to TNM stage M1. Approximately 8% of breast cancers are metastatic at the time of diagnosis, but up to 30% of patients will progress to metastatic disease [42]. Administered alone, or in combination with neoadjuvant chemotherapy, a calpain inhibitor might therefore prevent or slow progression to regional or distant metastasis. Notably, neoadjuvant chemotherapy itself can induce metastasis [43,44,45], and recent evidence suggests that needle biopsies may promote pro-metastatic changes within the tumor microenvironment by promoting epithelial-to-mesenchymal transition and tumor angiogenesis [46]. Thus, an effective calpain-1/2 inhibitor, administered together with neoadjuvant or adjuvant treatments, could improve clinical outcomes by suppressing metastasis. Based on the preclinical model data shown here, up to 80% of spontaneous metastasis could be prevented with effective calpain-1/2 inhibition. Calpain inhibition therefore has potential to extend the therapeutic window for conventional therapies before metastasis occurs. Additional studies are needed to explore whether calpain inhibition can abrogate chemotherapy-induced metastatic progression and, if so, in which clinical contexts.

The main roadblock preventing the exploitation of calpains as therapeutic targets is the lack of protease-specific cell-permeable inhibitors, and an insufficient understanding of the roles of different calpain isoforms in cancer. In this work, we address the second question by demonstrating that the inhibition of both calpain-1 and calpain-2 is necessary to achieve maximal suppression of metastasis. As discussed in recent reviews [8,35], isoform-specific roles of these calpain isoforms in the context of cancer have been reported. Our in vitro data using genetic models shows that two-dimensional migration is dependent, at least in part, on both calpain-1/2, which suggests non-redundant roles for these two calpain isoforms in this context. We have also demonstrated that inhibiting both isoforms is likely to be necessary to effectively abrogate in vivo metastasis. Again, this suggests that calpain-1 and calpain-2 may have overlapping roles in this context, and that their combined inhibition is required to achieve inhibition of metastasis in vivo.

The metastasis outcomes from the single-isoform knockouts highlight a more complex picture. *CAPN1* loss alone significantly reduced metastasis, whereas *CAPN2* loss did not, suggesting that calpain-1 activity may play a more prominent role in sustaining metastatic dissemination in this model. However, the rescue experiments revealed that restoring *CAPN1* expression did not significantly elevate metastasis above the KO level, while restoring *CAPN2* expression significantly increased metastasis compared to its KO. These findings point to possible differences in how each isoform contributes to metastatic potential when re-expressed in an established cell line, perhaps reflecting distinct substrate preferences or signaling pathway engagement, as seen in cancer previously [8]. Nevertheless, only the dual loss of both isoforms via *CAPNS1* KO yielded a consistent and profound suppression of metastasis, underscoring that therapeutic strategies targeting both calpain-1 and calpain-2 are likely required for maximum benefit.

One noteworthy limitation of our study is that all in vitro and in vivo experiments presented here were conducted only in the MDA-MB-231 cell line, a triple-negative breast cancer model. While this cell line is well-characterized and is highly metastatic, the observed interplay between the calpain-1 and calpain-2 isoforms may present differently in different contexts. However, the general role for calpain-1/2 in promoting metastasis has been established in several models [8,35], and thus, the differences are likely to manifest only in a relative importance of calpain-1 versus calpain-2. Yet, since no isoform-specific calpain inhibitors are commercially or clinically available at the moment, we suggest that the dual inhibition of both calpain-1 and calpain-2 may be more readily achievable and sufficient to inhibit metastasis. For models where specifically calpain-1 or calpain-2 is thought to play a dominant role in cancer progression, please refer to the literature [18,47,48,49]. Our genetic models explored cancer cell-intrinsic disruption of calpain-1/2, in contrast to a systemic mode of inhibition, likely to result from hypothetical future pharmaceutical approaches. This raises the concern of potential toxicities associated with systemic inhibition of calpains. Yet, we suggest that inhibiting calpain in an animal or in a human will not lead to unsurmountable acute adverse effects. Loss-of-function heritable *CAPN1* mutations have been described in human pedigrees [50,51,52]. While not lethal, these mutations are associated with late-onset ataxia, muscle wasting, and dystrophy [52,53]—severe phenotypes which are unlikely to arise from intermittent calpain inhibition. These phenotypes have also been observed in transgenic mice with germline knockout of *CAPN1* [52] or tissue-specific knockout of *CAPNS1* [53]. On the other hand, human pedigrees with *CAPN2* loss-of-function mutations have not been described, and germline *CAPN2* knockout is embryonically lethal in mice [54,55]. Similarly, germline *CAPNS1* knockout is also lethal [56], presumably through a lack of proteolytically active calpain-2. Despite this, tissue-specific or systemic disruption of *CAPNS1* in conditionally targeted mice is well tolerated [7,57,58,59,60,61,62], suggesting that transient pharmacological inhibition of calpain-1/2 in humans would not be acutely toxic.

Although CRISPR/Cas9 gene disruption of *CAPN1, CAPN2,* and *CAPNS1* in an engraftable cancer cell line model provided us with powerful tools for dissecting calpain-1 and calpain-2 contributions to metastasis, these approaches do not translate into therapeutics. Therefore, in our study, we also employed a membrane-permeable peptide inhibitor of calpain consisting of a peptide derived from calpastatin (CAST), which is believed to be specific for the calpain-1/2 isoforms [37,63]. This CAST B27 peptide was modified with nine R residues at the C-terminus to promote membrane penetration, several D-enantiomer amino acids at the N- and C-termini to protect it from exopeptidase activity, and an N-terminal fluorochrome to allow its detection in cells or in plasma. This modified B27 peptide (mB27) effectively inhibited purified recombinant calpain-2 in biochemical assays, and it penetrated cancer cells and inhibited cancer cell motility in vitro. The mB27 peptide had no effect on the migration behavior of *CAPNS1* KO cells, consistent with the conclusion that its effects were mediated by calpain inhibition. In vivo, in mice, the mB27 peptide exhibited a plasma half-life that was approximately an order of magnitude longer than expected for a similar all-L amino acid peptide (36 vs. 2–6 min [64,65], Figure 4f). This sub-optimal plasma half-life is perhaps the reason for a lack of a statistically significant response in the mB27 treatment cohort of mice compared to the vehicle control cohort. Nevertheless, the treatment group experienced a significantly lower variance in the extent of metastasis, with none of the ten mice displaying high levels of metastasis, compared to three of nine in the control cohort, suggesting that the sub-optimal calpain inhibition achieved in this study was perhaps successful at abrogating the occurrence of high levels of metastasis. Realizing the potential for a CAST-based peptide to become an effective therapeutic will require further work, but these observations provide incentive to pursue those efforts.

In summary, we have established that both calpain-1 and calpain-2 need to be inhibited in cancer cells to effectively prevent metastasis, and that the mB27 peptide used here worked well as an inhibitor in vitro but not in vivo. This provides justification for future drug discovery and development efforts against calpain-1 and calpain-2, aimed at producing inhibitors suitable for clinical use in cancer patients.

## Data Availability

All data described are contained within the article or available as Supplementary Information. The original images of lung metastasis presented in the study are openly available in the Zenodo repository at https://doi.org/10.5281/zenodo.16790973. Plasmids, proteins, cell lines, and mice can be made available upon request to I.Sh. or PAG.

## Supplementary Materials

The following supporting information can be downloaded at https://www.mdpi.com/article/10.3390/cells14171314/s1. Supplementary Information 1. CLUSTAL Omega sequence alignment of human CAPN1 and CAPN2; Supplementary Information 2. A summary of the CRISPR-Cas9 gene-targeting strategy; Supplementary Information 3. Oligonucleotides for Cas9-mediated calpain knockout; Supplementary Information 4. Primers used for RT/PCR cloning of human calpain cDNAs; Supplementary Information 5. Primers used for cloning calpain rescue constructs into pWPXLd; Supplementary Information 6. Anti-Cas9 immunizing mutations; Supplementary Information 7. Reagents for casein zymogram; Supplementary Information 8. Quantification of MSD and statistical analysis of calpain-deficient cell migration; Supplementary Information 9. Quantification and statistical analysis of tumor growth rates; Supplementary Information 10. Quantification and statistical analysis of metastasis; Supplementary Information 11. Calpain activity inhibited by an unmodified B-peptide of calpastatin; Supplementary Information 12. Quantification of MSD and statistical analysis of mB27-treated cells’ migration.

## References

[1] Walters S., Maringe C., Butler J., Rachet B., Barrett-Lee P., Bergh J., Boyages J., Christiansen P., Lee M., Wärnberg F. (2013). Breast cancer survival and stage at diagnosis in Australia, Canada, Denmark, Norway, Sweden and the UK, 2000–2007: A population-based study. Br. J. Cancer.

[2] Doyle C., Lohmann A.E., Iqbal N., Henning J.W., Kulkarni S., Califaretti N., Hilton J., Ferrario C., Bouganim N., Mates M. (2025). A Canadian real-world, multi-center, prospective, observational study assessing the treatment duration, the treatment sequence, and the overall survival for patients treated with endocrine therapy +/- targeted therapy in HR + HER2-negative advanced breast cancer. Breast Cancer Res. Treat..

[3] Zdenkowski N., Kuper-Hommel M.J.J., Niman S.M., Francis P.A., Baron-Hay S., Fox W., Menzies A.M., Angus R., Punie K., Zardawi S. (2025). Timing of nivolumab with neoadjuvant carboplatin and paclitaxel for early triple-negative breast cancer (BCT1902/IBCSG 61-20; Neo-N): A non-comparative, open-label, randomised, phase 2 trial. Lancet Oncol..

[4] Ellison L.F., Saint-Jacques N. (2023). Five-year cancer survival by stage at diagnosis in Canada. Health Rep..

[5] Ono Y., Sorimachi H. (2012). Calpains: An elaborate proteolytic system. Biochim. Biophys. Acta.

[6] Ono Y., Saido T.C., Sorimachi H. (2016). Calpain research for drug discovery: Challenges and potential. Nat. Rev. Drug Discov..

[7] Tan Y., Dourdin N., Wu C., De Veyra T., Elce J.S., Greer P.A. (2006). Conditional disruption of ubiquitous calpains in the mouse. Genesis.

[8] Storr S.J., Carragher N.O., Frame M.C., Parr T., Martin S.G. (2011). The calpain system and cancer. Nat. Rev. Cancer.

[9] Zhao C., Yuan G., Jiang Y., Xu J., Ye L., Zhan W., Wang J. (2020). Capn4 contributes to tumor invasion and metastasis in gastric cancer via activation of the Wnt/beta-catenin/MMP9 signalling pathways. Exp. Cell Res..

[10] Yu L.M., Zhu Y.S., Xu C.Z., Zhou L.L., Xue Z.X., Cai Z.Z. (2019). High calpain-1 expression predicts a poor clinical outcome and contributes to tumor progression in pancreatic cancer patients. Clin. Transl. Oncol..

[11] Storr S.J., Safuan S., Woolston C.M., Abdel-Fatah T., Deen S., Chan S.Y., Martin S.G. (2012). Calpain-2 expression is associated with response to platinum based chemotherapy, progression-free and overall survival in ovarian cancer. J. Cell Mol. Med..

[12] Tang S., Yin Q., Liu F., Zhang Y. (2019). Calpain Small Subunit 1 Protein in the Prognosis of Cancer Survivors and Its Clinicopathological Correlation. Biomed. Res. Int..

[13] Chen J., Wu Y., Zhang L., Fang X., Hu X. (2019). Evidence for calpains in cancer metastasis. J. Cell Physiol..

[14] Madeira F., Madhusoodanan N., Lee J., Eusebi A., Niewielska A., Tivey A.R.N., Lopez R., Butcher S. (2024). The EMBL-EBI Job Dispatcher sequence analysis tools framework in 2024. Nucleic Acids Res..

[15] Strobl S., Fernandez-Catalan C., Braun M., Huber R., Masumoto H., Nakagawa K., Irie A., Sorimachi H., Bourenkow G., Bartunik H. (2000). The crystal structure of calcium-free human m-calpain suggests an electrostatic switch mechanism for activation by calcium. Proc. Natl. Acad. Sci. USA.

[16] Abramson J., Adler J., Dunger J., Evans R., Green T., Pritzel A., Ronneberger O., Willmore L., Ballard A.J., Bambrick J. (2024). Accurate structure prediction of biomolecular interactions with AlphaFold 3. Nature.

[17] Liao H.J., Carpenter G. (2012). Regulated intramembrane cleavage of the EGF receptor. Traffic.

[18] Kulkarni S., Reddy K.B., Esteva F.J., Moore H.C., Budd G.T., Tubbs R.R. (2010). Calpain regulates sensitivity to trastuzumab and survival in HER2-positive breast cancer. Oncogene.

[19] Kulkarni S., Goll D.E., Fox J.E. (2002). Calpain cleaves RhoA generating a dominant-negative form that inhibits integrin-induced actin filament assembly and cell spreading. J. Biol. Chem..

[20] Franco S.J., Rodgers M.A., Perrin B.J., Han J., Bennin D.A., Critchley D.R., Huttenlocher A. (2004). Calpain-mediated proteolysis of talin regulates adhesion dynamics. Nat. Cell Biol..

[21] Chan K.T., Bennin D.A., Huttenlocher A. (2010). Regulation of adhesion dynamics by calpain-mediated proteolysis of focal adhesion kinase (FAK). J. Biol. Chem..

[22] Carragher N.O., Walker S.M., Scott Carragher L.A., Harris F., Sawyer T.K., Brunton V.G., Ozanne B.W., Frame M.C. (2006). Calpain 2 and Src dependence distinguishes mesenchymal and amoeboid modes of tumour cell invasion: A link to integrin function. Oncogene.

[23] Jeon K.H., Park S., Pak E.S., Kim J.A., Liu Y., Hwang S.Y., Na Y., Kwon Y. (2025). Calpain 2 Isoform-Specific Cleavage of Filamin A Enhances HIF1α Nuclear Translocation, Promoting Metastasis in Triple-Negative Breast Cancer. MedComm.

[24] Rios-Doria J., Day K.C., Kuefer R., Rashid M.G., Chinnaiyan A.M., Rubin M.A., Day M.L. (2003). The role of calpain in the proteolytic cleavage of E-cadherin in prostate and mammary epithelial cells. J. Biol. Chem..

[25] Conacci-Sorrell M., Ngouenet C., Anderson S., Brabletz T., Eisenman R.N. (2014). Stress-induced cleavage of Myc promotes cancer cell survival. Genes. Dev..

[26] Gao G., Dou Q.P. (2000). N-terminal cleavage of bax by calpain generates a potent proapoptotic 18-kDa fragment that promotes bcl-2-independent cytochrome C release and apoptotic cell death. J. Cell Biochem..

[27] Goll D.E., Thompson V.F., Li H., Wei W., Cong J. (2003). The calpain system. Physiol. Rev..

[28] Ackermann A., Brieger A. (2019). The Role of Nonerythroid Spectrin αII in Cancer. J. Oncol..

[29] Sanjana N.E., Shalem O., Zhang F. (2014). Improved vectors and genome-wide libraries for CRISPR screening. Nat. Methods.

[30] Croall D.E., Moffett K., Hatch H. (2002). Casein zymography of calpains using a 4-(2-hydroxyethyl)-1-piperazineethanesulfonic acid-imidazole buffer. Anal. Biochem..

[31] Gorelik R., Gautreau A. (2014). Quantitative and unbiased analysis of directional persistence in cell migration. Nat. Protoc..

[32] Grieve S., Gao Y., Hall C., Hu J., Greer P.A. (2016). Calpain Genetic Disruption and HSP90 Inhibition Combine To Attenuate Mammary Tumorigenesis. Mol. Cell Biol..

[33] Colucci F., Soudais C., Rosmaraki E., Vanes L., Tybulewicz V.L., Di Santo J.P. (1999). Dissecting NK cell development using a novel alymphoid mouse model: Investigating the role of the c-abl proto-oncogene in murine NK cell differentiation. J. Immunol..

[34] O’Clair L., Shean J., Kolozsvari B. (2017). Live-Cell Analysis Handbook: A Guide to Real-Time Live-Cell Imaging and Analysis.

[35] Shapovalov I., Harper D., Greer P.A. (2022). Calpain as a therapeutic target in cancer. Expert. Opin. Ther. Targets.

[36] Harper D., Min J.Y., MacLeod J.A., Cockburn S., Predko I., Gao Y., Greer P., Shapovalov I. (2025). Dataset Related to Article: “Calpain-1 and Calpain-2 Promote Breast Cancer Metastasis” (Version 1). Zenodo. https://zenodo.org/records/16790974.

[37] Hanna R.A., Campbell R.L., Davies P.L. (2008). Calcium-bound structure of calpain and its mechanism of inhibition by calpastatin. Nature.

[38] Pfizer J., Assfalg-Machleidt I., Machleidt W., Schaschke N. (2008). Inhibition of human mu-calpain by conformationally constrained calpastatin peptides. Biol. Chem..

[39] Jin J., Wu Y., Chen J., Shen Y., Zhang L., Zhang H., Chen L., Yuan H., Chen H., Zhang W. (2020). The peptide PROTAC modality: A novel strategy for targeted protein ubiquitination. Theranostics.

[40] McCartney C.E., MacLeod J.A., Greer P.A., Davies P.L. (2018). An easy-to-use FRET protein substrate to detect calpain cleavage in vitro and in vivo. Biochim. Biophys. Acta Mol. Cell Res..

[41] Huttenlocher A., Palecek S.P., Lu Q., Zhang W., Mellgren R.L., Lauffenburger D.A., Ginsberg M.H., Horwitz A.F. (1997). Regulation of cell migration by the calcium-dependent protease calpain. J. Biol. Chem..

[42] O’Shaughnessy J. (2005). Extending survival with chemotherapy in metastatic breast cancer. Oncologist.

[43] Karagiannis G.S., Pastoriza J.M., Wang Y., Harney A.S., Entenberg D., Pignatelli J., Sharma V.P., Xue E.A., Cheng E., D’Alfonso T.M. (2017). Neoadjuvant chemotherapy induces breast cancer metastasis through a TMEM-mediated mechanism. Sci. Transl. Med..

[44] Hoskin V., Ghaffari A., Laight B.J., SenGupta S., Madarnas Y., Nicol C.J.B., Elliott B.E., Varma S., Greer P.A. (2022). Targeting the Ezrin Adaptor Protein Sensitizes Metastatic Breast Cancer Cells to Chemotherapy and Reduces Neoadjuvant Therapy-induced Metastasis. Cancer Res. Commun..

[45] Volmer L., Koch A., Matovina S., Dannehl D., Weiss M., Welker G., Hahn M., Engler T., Wallwiener M., Walter C.B. (2022). Neoadjuvant Chemotherapy of Patients with Early Breast Cancer Is Associated with Increased Detection of Disseminated Tumor Cells in the Bone Marrow. Cancers.

[46] Kameyama H., Dondapati P., Simmons R., Leslie M., Langenheim J.F., Sun Y., Yi M., Rottschaefer A., Pathak R., Nuguri S. (2023). Needle biopsy accelerates pro-metastatic changes and systemic dissemination in breast cancer: Implications for mortality by surgery delay. Cell Rep. Med..

[47] Gao X., Mao Y.H., Xiao C., Li K., Liu W., Li L.Y., Pang J. (2018). Calpain-2 triggers prostate cancer metastasis via enhancing CRMP4 promoter methylation through NF-kappaB/DNMT1 signaling pathway. Prostate.

[48] Hossain M.I., Roulston C.L., Kamaruddin M.A., Chu P.W., Ng D.C., Dusting G.J., Bjorge J.D., Williamson N.A., Fujita D.J., Cheung S.N. (2013). A truncated fragment of Src protein kinase generated by calpain-mediated cleavage is a mediator of neuronal death in excitotoxicity. J. Biol. Chem..

[49] Cortesio C.L., Chan K.T., Perrin B.J., Burton N.O., Zhang S., Zhang Z.-Y., Huttenlocher A. (2008). Calpain 2 and PTP1B function in a novel pathway with Src to regulate invadopodia dynamics and breast cancer cell invasion. J. Cell Biol..

[50] Lai L.L., Chen Y.J., Li Y.L., Lin X.H., Wang M.W., Dong E.L., Wang N., Chen W.J., Lin X. (2020). Novel CAPN1 mutations extend the phenotypic heterogeneity in combined spastic paraplegia and ataxia. Ann. Clin. Transl. Neurol..

[51] Kim A., Kumar K.R., Davis R.L., Mallawaarachchi A.C., Gayevskiy V., Minoche A.E., Walls Z., Kim H.J., Jang M., Cowley M.J. (2019). Increased Diagnostic Yield of Spastic Paraplegia with or Without Cerebellar Ataxia Through Whole-Genome Sequencing. Cerebellum.

[52] Wang Y., Hersheson J., Lopez D., Hammer M., Liu Y., Lee K.-H., Pinto V., Seinfeld J., Wiethoff S., Sun J. (2016). Defects in the CAPN1 Gene Result in Alterations in Cerebellar Development and Cerebellar Ataxia in Mice and Humans. Cell Rep..

[53] Piper A.K., Sophocleous R.A., Ross S.E., Evesson F.J., Saleh O., Bournazos A., Yasa J., Reed C., Woolger N., Sluyter R. (2020). Loss of calpains-1 and -2 prevents repair of plasma membrane scrape injuries, but not small pores, and induces a severe muscular dystrophy. Am. J. Physiol. Cell Physiol..

[54] Takano J., Mihira N., Fujioka R., Hosoki E., Chishti A.H., Saido T.C. (2011). Vital Role of the Calpain-Calpastatin System for Placental-Integrity-Dependent Embryonic Survival. Mol. Cell. Biol..

[55] Dutt P., Croall D.E., Arthur J.S., Veyra T.D., Williams K., Elce J.S., Greer P.A. (2006). m-Calpain is required for preimplantation embryonic development in mice. BMC Dev. Biol..

[56] Zimmerman U.J., Boring L., Pak J.H., Mukerjee N., Wang K.K. (2000). The calpain small subunit gene is essential: Its inactivation results in embryonic lethality. IUBMB Life.

[57] Shimada M., Greer P.A., McMahon A.P., Bouxsein M.L., Schipani E. (2008). In vivo targeted deletion of calpain small subunit, Capn4, in cells of the osteoblast lineage impairs cell proliferation, differentiation, and bone formation. J. Biol. Chem..

[58] Amini M., Ma C.L., Farazifard R., Zhu G., Zhang Y., Vanderluit J., Zoltewicz J.S., Hage F., Savitt J.M., Lagace D.C. (2013). Conditional disruption of calpain in the CNS alters dendrite morphology, impairs LTP, and promotes neuronal survival following injury. J. Neurosci..

[59] Kashiwagi A., Schipani E., Fein M.J., Greer P.A., Shimada M. (2010). Targeted deletion of Capn4 in cells of the chondrocyte lineage impairs chondrocyte proliferation and differentiation. Mol. Cell Biol..

[60] Ni R., Zheng D., Xiong S., Hill D.J., Sun T., Gardiner R.B., Fan G.C., Lu Y., Abel E.D., Greer P.A. (2016). Mitochondrial Calpain-1 Disrupts ATP Synthase and Induces Superoxide Generation in Type 1 Diabetic Hearts: A Novel Mechanism Contributing to Diabetic Cardiomyopathy. Diabetes.

[61] Wernimont S.A., Simonson W.T., Greer P.A., Seroogy C.M., Huttenlocher A. (2010). Calpain 4 is not necessary for LFA-1-mediated function in CD4+ T cells. PLoS ONE.

[62] Yang J., Xiang F., Cai P.C., Lu Y.Z., Xu X.X., Yu F., Li F.Z., Greer P.A., Shi H.Z., Zhou Q. (2016). Activation of calpain by renin-angiotensin system in pleural mesothelial cells mediates tuberculous pleural fibrosis. Am. J. Physiol. Lung Cell Mol. Physiol..

[63] Wendt A., Thompson V.F., Goll D.E. (2004). Interaction of calpastatin with calpain: A review. Biol. Chem..

[64] Hong S.Y., Oh J.E., Lee K.H. (1999). Effect of D-amino acid substitution on the stability, the secondary structure, and the activity of membrane-active peptide. Biochem. Pharmacol..

[65] Diao L., Meibohm B. (2013). Pharmacokinetics and pharmacokinetic-pharmacodynamic correlations of therapeutic peptides. Clin. Pharmacokinet..

