# Supplementary material for: Calpain-1 and Calpain-2 Promote Breast Cancer Metastasis
Source: Cells. 2025 Aug 25;14(17):1314. doi: 10.3390/cells14171314 (PMC12427806; doi:10.3390/cells14171314)
Supplement: Supplementary file 1 [file cells-14-01314-s001.zip › cells-3797288-supplementary.pdf]

Title

Calpain-1 and calpain-2 promote breast cancer metastasis

Authors

Danielle Harper[1,2], Jung Yeon Min[1,2], James A. MacLeod[1,2], Samantha Cockburn[1,2], Iryna Predko[1,2], Yan Gao[1,2], Peter A. Greer[1,2], and Ivan Shapovalov[1,2,*]

[1] Dept Pathology and Molecular Medicine, Queen's University, Kingston, ON, Canada, K7L3N6; danielle.harper@queensu.ca, jungyeon.min@mail.utoronto.ca, james.macleod@queensu.ca, samantha2cockburn@gmail.com, iren.predko@gmail.com, yg2@queensu.ca, greerp@queensu.ca, ivan.shapovalov@queensu.ca

[2] Division of Cancer Biology and Genetics, Sinclair Cancer Research Institute, Queen's University, Kingston, ON, Canada, K7L3N6

* Correspondence: ivan.shapovalov@queensu.ca

List of the material included

Supplementary Information 1. CLUSTAL Omega sequence alignment of human CAPN1 and CAPN2. **Page S-2**
Supplementary Information 2. A summary of the CRISPR-Cas9 gene-targeting strategy. **Page S-3**
Supplementary Information 3. Oligonucleotides for Cas9-mediated calpain knockout. **Page S-4**
Supplementary Information 4. Primers used for RT/PCR cloning of human calpain cDNAs. **Page S-4**
Supplementary Information 5. Primers used for cloning calpain rescue constructs into pWPXLd. **Page S-5**
Supplementary Information 6. Anti-Cas9 immunizing mutations. **Page S-5**
Supplementary Information 7. Reagents for casein zymogram. **Page S-6**
Supplementary Information 8. Quantification of MSD and statistical analysis of calpain-deficient cell migration. **Page S-8**
Supplementary Information 9. Quantification and statistical analysis of tumor growth rates. **Page S-8**
Supplementary Information 10. Quantification and statistical analysis of metastasis. **Page S-9**
Supplementary Information 11. Calpain activity inhibited by an unmodified B-peptide of calpastatin. **Page S-9**
Supplementary Information 12. Quantification of MSD and statistical analysis of mB27-treated cells' migration. **Page S-10**

Supplementary Information 1. CLUSTAL Omega sequence alignment of human CAPN1 and CAPN2.

```
CAPN1      MSEEIITPVYCTGVSAQVQKQRARELGLGRHENAIKYLGQDYEQLRVRCLQSGTLFRDEA     60
CAPN2      ----------MAGIAAKLAKDREAAEGLGSHDRAIKYLNQDYEALRNECLEAGTLFQDPS     50
                      :*::*:: *:*    *** *:.*****.**** ** .**::****:* :

CAPN1      FPPVPQSLGYKDLGPNSSKTYGIKWKRPTELLSNPQFIVDGATRTDICQGALGDCWLLAA     120
CAPN2      FPAIPSALGFKELGPYSSKTRGIEWKRPTEICADPQFIIGGATRTDICQGALGDCWLLAA     110
           ** :*.:**:*:*** **** **:******: ::*****:.*******************

CAPN1      IASLTLNDTLLHRVVPHGQSFQNGYAGIFHFQLWQFGEWVDVVVDDLLPIKDGKLVFVHS     180
CAPN2      IASLTLNEEILARVVPLNQSFQENYAGIFHFQFWQYGEWVEVVVDDRLPTKDGELLFVHS     170
           *******: :* **** .****:.********:**:****:***** ** ***:*:****

CAPN1      AEGNEFWSALLEKAYAKVNGSYEALSGGSTSEGFEDFTGGVTEWYELRKAPSDLYQIILK     240
CAPN2      AEGSEFWSALLEKAYAKINGCYEALSGGATTEGFEDFTGGIAEWYELKKPPPNLFKIIQK     230
           ***.*************:**.*******:*:*********::*****:* * :*::** *

CAPN1      ALERGSLLGCSIDISSVLDMEAITFKKLVKGHAYSVTGAKQVNYRGQVVSLIRMRNPWGE     300
CAPN2      ALQKGSLLGCSIDITSAADSEAITFQKLVKGHAYSVTGAEEVESNGSLQKLIRIRNPWGE     290
           **::**********:*. * *****:*************::*: .*.: .***:******

CAPN1      VEWTGAWSDSSSEWNNVDPYERDQLRVKMEDGEFWMSFRDFMREFTRLEICNLTPDALKS     360
CAPN2      VEWTGRWNDNCPSWNTIDPEERERLTRRHEDGEFWMSFSDFLRHYSRLEICNLTPDTLTS     350
           ***** *.*.. .**.:** **::*  : ********* **:*.::**********:*.*

CAPN1      RTIRKWNTTLYEGTWRRGSTAGGCRNYPATFWVNPQFKIRLDETDDPDDYGDRESGCSFV     420
CAPN2      DTYKKWKLTKMDGNWRRGSTAGGCRNYPNTFWMNPQYLIKLEEEDEDE--EDGESGCTFL     408
            * :**: *  :*.************** ***:***: *:*:* *: :   * ****:*:

CAPN1      LALMQKHRRRERRFGRDMETIGFAVYEVPPELVGQPAVHLKRDFFLANASRARSEQFINL     480
CAPN2      VGLIQKHRRRQRKMGEDMHTIGFGIYEVPEELSGQTNIHLSKNFFLTNRARERSDTFINL     468
           :.*:******:*::*.**.****.:**** ** **  :**.::***:* :* **: ****

CAPN1      REVSTRFRLPPGEYVVVPSTFEPNKEGDFVLRFFSEKSAGTVELDDQIQANLPDEQVLSE     540
CAPN2      REVLNRFKLPPGEYILVPSTFEPNKDGDFCIRVFSEKKADYQAVDDEIEANLEEF-DISE     527
           *** .**:******::*********:*** :*.****.*.   :**:*:*** :   :**

CAPN1      EEIDENFKALFRQLAGEDMEISVKELRTILNRIISKHKDLRTKGFSLESCRSMVNLMDRD     600
CAPN2      DDIDDGFRRLFAQLAGEDAEISAFELQTILRRVLAKRQDIKSDGFSIETCKIMVDMLDSD     587
           ::**:.*: ** ****** ***. **:***.*:::*::*:::.***:*:*: **:::* *

CAPN1      GNGKLGLVEFNILWNRIRNYLSIFRKFDLDKSGSMSAYEMRMAIESAGFKLNKKLYELII     660
CAPN2      GSGKLGLKEFYILWTKIQKYQKIYREIDVDRSGTMNSYEMRKALEEAGFKMPCQLHQVIV     647
           *.***** ** ***.:*::* .*:*::*:*:**:*.:**** *:*.****:  :*:::*:

CAPN1      TRYSEPDLAVDFDNFVCCLVRLETMFRFFKTLDTDLDGVVTFDLFKWLQLTMFA  714
CAPN2      ARFADDQLIIDFDNFVRCLVRLETLFKIFKQLDPENTGTIELDLISWLCFSVL-  700
           :*::: :* :****** *******:*::** ** :  *.: :**:.** ::::
```

*—exact identity (439 amino acids).

:—strongly conserved residues (140 amino acids).

.—weakly conserved residues (41 amino acids).

Blank—no similarity (91 amino acids).

Supplementary Information 2. A summary of the CRISPR-Cas9 gene-targeting strategy.

CAPNS1 is a gene encoded on chromosome 19, between nucleotides 36,140,066 and 36,150,353. The gene encodes 11 exons. In accordance with the requirements for an optimal sgRNA sequence for use with the lentiCRIPSRv2 [29] CRISPR-Cas9 KO system, we chose nucleotides 36142303 to 36142325 as a template for the sgRNA (including the PAM sequence), which encodes Ala72 to Pro78 of CAPNS1 in exon 3. For the subsequent addback rescue construct, we chose to introduce a silent mutation CCA into the Pro78 codon CCG, which would immunize it against the CRISPR-Cas9. The exact primer sequences for the cloning of the lentiCRIPSRv2 sgRNA, for PCR amplification of the CAPNS1 cDNA, and for the site-directed mutagenesis are shown in Supplementary Information 2-5.

CAPN1 is a gene encoded on chromosome 11, between nucleotides 65,181,373 and 65,212,006. The gene encodes 22 exons. In accordance with the requirements for an optimal sgRNA sequence for use with the lentiCRIPSRv2 [29] CRISPR-Cas9 KO system, we chose nucleotides 65182924 to 65212006 as a template for the sgRNA (including the PAM sequence), which encodes Pro75 to Gly82 of CAPN1 in exon 2. For the subsequent addback rescue construct, we chose to introduce a silent mutation AGTAGT into the Ser77-Ser78 codons TCCTCC, which would immunize it against the CRISPR-Cas9. The exact primer sequences for the cloning of the lentiCRIPSRv2 sgRNA, for PCR amplification of the CAPN1 cDNA, and for the site-directed mutagenesis are shown in Supplementary Information 2-5.

CAPN2 is a gene encoded on chromosome 1, between nucleotides 223,712,539 and 223,776,018. The gene encodes 21 exons. In accordance with the requirements for an optimal sgRNA sequence for use with the lentiCRIPSRv2 [29] CRISPR-Cas9 KO system, we chose nucleotides 223745388 to 223745410 as a template for the sgRNA (including the PAM sequence), which encodes Ser92 to Trp98 of CAPN2 in exon 4. For the subsequent addback rescue construct, we chose to introduce a silent mutation GGTTCT into the Gly95-Ser96 codons GGGAGC, which would immunize it against the CRISPR-Cas9. The exact primer sequences for the cloning of the lentiCRIPSRv2 sgRNA, for PCR amplification of the CAPN2 cDNA, and for the site-directed mutagenesis are shown in Supplementary Information 2-5.

Supplementary Information 3. Oligonucleotides for Cas9-mediated calpain knockout.

Sequences contain 20-mers homologous to target sequences and sticky ends for cloning into BsmBI site of lentiCRISPRv2 plasmid. Sequences identical to + strand of the target are highlighted.

Target	Sequence
CAPN1, exon 2	Oligo 1:
	5′- CACCG CCCAATTCCTCCAAGACCTA - 3′
	Oligo 2:
	3′ - C GGGTTAAGGAGGTTCTGGAT CAAA - 5′
CAPN2, exon 3	Oligo 1:
	5′- CACCG CAGCCGAAGGGAGCGAGTTC - 3′
	Oligo 2:
	3′ - C GTCGGCTTCCCTCGCTCAAG CAAA - 5′
CAPNS1, exon 3	Oligo 1:
	5′- CACCG GGCGGCTGCGCAGTACAACC- 3′
	Oligo 2:
	3′ - C CCGCCGACGCGTCATGTTGG CAAA - 5′

Supplementary Information 4. Primers used for RT/PCR cloning of human calpain cDNAs.

Oligonucleotides contain sequences homologous to the targets and an NcoI restriction site (5'-CCATGG-3') for cloning into the same site in the pCS2-MT expression plasmid to produce N-terminally Myc-epitope-tagged recombinant proteins.

Insert	Sequence
CAPN1	Fw: 5′ – ACCCCTCCATGGGGATGTCGGAGGAGATCATCACGCC – 3′
	Rv: 5′ – CGAGTCCCATGGTCATGCAAACATGGTCAGCTGCAACC – 3′
CAPN2	Fw: 5′ – GCCGGGCCATGGGCATGGCGGGCATCGCGGCCAAGC – 3′
	Rv: 5′ – TTAGTTCCATGGTCAAAGTACTGAGAAACAGAGCCAAG – 3′
CAPNS1	Fw: 5′ – GCGGTGCCATGGGAACGGCCATGCGCATCCTAGGCGG – 3′
	Rv: 5′ – TTAACCCCATGGTCAGGAATACATAGTCAGCTGCAGCC – 3′

Supplementary Information 5. Primers used for cloning calpain rescue constructs into pWPXLd.

Oligonucleotides for CAPN1 and CAPNS1 calpain constructs target regions of pCS2-MT plasmid to incorporate the Myc-tag upon shuttle-cloning.

Insert	Sequence
CAPN1/S1	Fw: 5′ – CTTTTTGTTTAAACCCATCGATTTAAAGCTATGGAGCAA– 3′
	Rv: 5′ – TTTGTCGTTTAAACTCAATGTATCTTATCATGTCTGGAT – 3′
CAPN2	Fw: 5′ –CTTTTTGTTTAAACATGGCGGGCATCGCGGCCAAGCTGG– 3′
	Rv: 5′ –TTTGTCGTTTAAACTCAAAGTACTGAGAAACAGAGCCAA – 3′

Supplementary Information 6. Anti-Cas9 immunizing mutations.

Mutated nucleotides are highlighted in bold. Nucleotides are numbered starting from adenine residue in the start codon.

Insert	Sequence
CAPN1, nucl.223-245	Before: 5’- CCCAATTCCTCCAAGACCTATGG - 3’
	After: 5’- CCCAAT**AGTAGT**AAGACCTATGG - 3’
CAPN2,	Before: 5’- CAGCCGAAGGGAGCGAGTTC - 3’
nucl.509-528	After: 5’- CAGCCGAAGG**TTCT**GAGTTC - 3’
CAPNS1, nucl.213-235	Before: 5’- GGCGGCTGCGCAGTACAACCCGG - 3’
	After: 5’- GGCGGCTGCGCAGTACAACCC**A**G - 3’

Supplementary Information 7. Reagents for casein zymogram.

Reagent	Composition<br>Compound (final conc.)—amount required
SDS Coomassie Stain	Coomassie brilliant blue G250—2 g
	Methanol—400 mL
	Acetic acid—100 mL
	Add dH2O to 1 L.
	Filter with Whatman paper.
SDS Destaining Solution	dH2O—700 mL
	Methanol—200 mL
	Acetic acid—100 mL
Lysis Buffer	HEPES (50 mM)—5.96 g
	NaCl (150 mM)—4.38 g
	Glycerol (10%)—50 mL
	Triton X-100 (1%)—5 mL
	EDTA (5 mM)—0.93 g
	Bring to 500 mL with dH2O.
	Prior to use, add
	2-mercapthoethanol (2ME)—1 uL / 1 mL LB
	PMSF—10 uL / 1 mL LB
	Leupeptin—1 uL / 1mL LB
5X HI Buffer Stock	Imidazole—7.32 g
	HEPES—20.85 g
	Bring to 500 mL with dH2O.
	pH=7.4. Do not adjust.
Casein	Casein—10 mg/mL in 2x HI buffer
	Make 10 mL.
	Add 0.5 uL 2ME/10mL.
	Stir for 20-30 mins before use. Make fresh daily.
2x Sample Buffer	Tris-HCl buffer—2mL of 0.5M
	EDTA—0.2 mL of 0.5M
	Glycerol—0.2 mL of 50%
	Bromophenol blue—20 mg

	2ME—0.2 mL
	dH2O—4 mL
Development Buffer	Tris—0.91 g
	MOPS—1.57 g
	5X HI buffer—30 mL
	CaCl2—0.22 g
	2ME—300 uL
	Bring to 300 uL with dH2O.
Running Gel	30% acrylamide—3.33 mL
	5X HI buffer—1.2 mL
	10mg/mL casein—2 ml
	0.2 M EGTA—0.1 mL
	dH2O—3.3 mL
Stacking Gel	30% acrylamide—0.8 mL
	5X HI buffer—1 mL
	H2O—3.2 mL
Running Buffer	5X HI—200 mL
	dH2O—800 mL
	0.5M EDTA—1.25 mL
	2ME—1 mL

Supplementary Information 8. Quantification of MSD and statistical analysis of calpain-deficient cell migration.

	MDA-MB-231 (WT)	CAPN1 KO	CAPN1 Rescue	CAPN2 KO	CAPN2 Rescue	CAPNS1 KO	CAPNS1 Rescue
Best-fit values							
Slope	0.006504	0.0004315	0.007483	0.0003838	0.005654	0.0007218	0.007917
Std. Error							
Slope	0.0006742	3.671e-005	0.0009328	3.196e-005	0.0006958	7.774e-005	0.0008004
Is slope significantly non-zero?							
F	93.06	138.2	64.35	144.2	66.03	86.20	97.84
DFn, DFd	1, 658	1, 658	1, 658	1, 658	1, 658	1, 658	1, 658
P value	<0.0001	<0.0001	<0.0001	<0.0001	<0.0001	<0.0001	<0.0001
Deviation from zero?	Significant	Significant	Significant	Significant	Significant	Significant	Significant

Supplementary Information 9. Quantification and statistical analysis of tumor growth rates.

ANOVA summary	
F	5.419
P value	<0.0001
P value summary	****
Significant diff. among means (P < 0.05)?	Yes

Uncorrected Fisher's LSD	Mean Diff.	95.00% CI of diff.	Summary	Individual P Value
WT vs. CAPN1 KO	0.05444	-0.06466 to 0.1735	ns	0.3663
WT vs. CAPN1 R	0.1599	0.04080 to 0.2790	**	0.0091
WT vs. CAPN2 KO	-0.04068	-0.1489 to 0.06753	ns	0.4572
WT vs. CAPN2 R	0.08980	-0.01101 to 0.1906	ns	0.0802
WT vs. CAPNS1 KO	0.2199	0.1008 to 0.3390	***	0.0004
WT vs. CAPNS1 R	-0.1236	-0.2476 to 0.0004121	ns	0.0508
CAPN1 KO vs. CAPN1 R	0.1055	-0.04100 to 0.2519	ns	0.1561
CAPN1 KO vs. CAPN2 KO	-0.09512	-0.2329 to 0.04263	ns	0.1736
CAPN1 KO vs. CAPN2 R	0.03536	-0.09666 to 0.1674	ns	0.5961
CAPN1 KO vs. CAPNS1 KO	0.1655	0.01904 to 0.3120	*	0.0272
CAPN1 KO vs. CAPNS1 R	-0.1780	-0.3285 to -0.02756	*	0.0209
CAPN1 R vs. CAPN2 KO	-0.2006	-0.3383 to -0.06283	**	0.0048
CAPN1 R vs. CAPN2 R	-0.07010	-0.2021 to 0.06192	ns	0.2944
CAPN1 R vs. CAPNS1 KO	0.06004	-0.08642 to 0.2065	ns	0.4176
CAPN1 R vs. CAPNS1 R	-0.2835	-0.4340 to -0.1330	***	0.0003
CAPN2 KO vs. CAPN2 R	0.1305	0.008196 to 0.2528	*	0.0368
CAPN2 KO vs. CAPNS1 KO	0.2606	0.1229 to 0.3984	***	0.0003
CAPN2 KO vs. CAPNS1 R	-0.08291	-0.2249 to 0.05910	ns	0.2492
CAPN2 R vs. CAPNS1 KO	0.1301	-0.001876 to 0.2622	ns	0.0533
CAPN2 R vs. CAPNS1 R	-0.2134	-0.3498 to -0.07693	**	0.0025
CAPNS1 KO vs. CAPNS1 R	-0.3435	-0.4940 to -0.1931	****	<0.0001

Descr. Statistic	WT	CAPN1 KO	CAPN1 R	CAPN2 KO	CAPN2 R	CAPNS1 KO	CAPNS1 R
Number of values	31	10	10	13	16	10	9
Mean	1.000	0.9456	0.8401	1.041	0.9102	0.7801	1.124
Std. Deviation	0.1677	0.2254	0.1235	0.1795	0.1493	0.1457	0.1373
Std. Error of Mean	0.03012	0.07126	0.03904	0.04980	0.03732	0.04608	0.04578
Lower 95% CI	0.9385	0.7844	0.7518	0.9322	0.8306	0.6758	1.018
Upper 95% CI	1.062	1.107	0.9284	1.149	0.9898	0.8843	1.229

Supplementary Information 10. Quantification and statistical analysis of metastasis.

ANOVA summary	
F	6.294
P value	<0.0001
P value summary	****
Significant diff. among means (P < 0.05)?	Yes

Uncorrected Fisher's LSD	Mean Diff.	95.00% CI of diff.	Summary	Individual P Value
WT vs. CAPN1 KO	0.7677	0.2579 to 1.277	**	0.0037
CAPN1 KO vs. CAPN1 R	-0.4150	-1.076 to 0.2456	ns	0.2148
WT vs. CAPN2 KO	0.2488	-0.2032 to 0.7008	ns	0.2765
WT vs. CAPNS1 KO	0.8344	0.3474 to 1.322	**	0.0010
CAPN2 KO vs. CAPN2 R	-0.7251	-1.269 to -0.1808	**	0.0097
CAPNS1 KO vs. CAPNS1 R	-1.260	-1.862 to -0.6585	****	<0.0001

Descr. Statistic	WT	CAPN1 KO	CAPN1 R	CAPN2 KO	CAPN2 R	CAPNS1 KO	CAPNS1 R
Number of values	29	8	7	11	11	9	9
Mean	1.000	0.2323	0.6474	0.7512	1.476	0.1656	1.426
Std. Deviation	0.5583	0.2048	0.5450	0.6496	1.182	0.1359	0.5593
Std. Error of Mean	0.1037	0.07242	0.2060	0.1959	0.3564	0.04532	0.1864
Lower 95% CI	0.7876	0.06109	0.1433	0.3148	0.6822	0.06106	0.9959
Upper 95% CI	1.212	0.4036	1.151	1.188	2.270	0.2701	1.856

Supplementary Information 11. Calpain activity inhibited by an unmodified B-peptide of calpastatin.

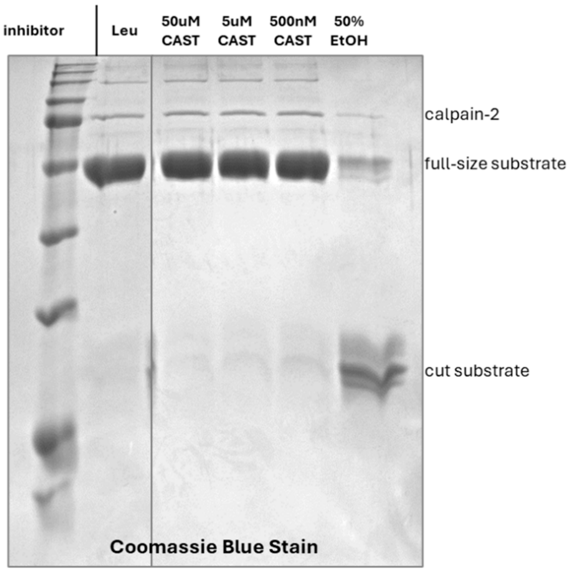

Leu: excess leupeptin
CAST: DPMSSTYIEELGKREVTIPPKYRELLA
50% EtOH: vehicle control

Supplementary Information 12. Quantification of MSD and statistical analysis of mB27-treated cells' migration.

	WT + vehicle	WT + mB27	CAPNS1 KO + vehicle	CAPNS1 KO + mB27
Best-fit values				
Slope	14.65	7.652	3.642	3.365
Std. Error				
Slope	1.498	0.6565	0.3214	0.4558
Is slope significantly non -zero?				
F	95.71	135.8	128.4	54.49
DFn, DFd	1, 678	1, 678	1, 712	1, 644
P value	<0.0001	<0.0001	<0.0001	<0.0001
Deviation from zero?	Significant	Significant	Significant	Significant
